# Missed opportunities for maternal immunisation against influenza and COVID-19, Norway, October 2023 to May 2024: a population-based registry study

**DOI:** 10.2807/1560-7917.ES.2026.31.7.2500504

**Published:** 2026-02-19

**Authors:** Melanie Stecher, Margrethe Greve-Isdahl, Jesper Dahl, Evy Dvergsdal, Suzanne Campbell, Frederik Skår, Svein Rune Andersen, Kjersti Margrethe Rydland, Inger Johanne Bakken, Hilde Marie Engjom, Hinta Meijerink

**Affiliations:** 1 Department of Infection Control and Vaccines, Norwegian Institute of Public Health, Oslo, Norway; 2 Faculty of Medicine, University of Oslo, Oslo, Norway; 3 Department of Infection Control Registries, Norwegian Institute of Public Health, Oslo, Norway; 4 Department of Chronic Diseases, Norwegian Institute of Public Health, Oslo, Norway; 5 Department of Health Registry Research and Development, Norwegian Institute of Public Health, Bergen, Norway

**Keywords:** maternal vaccination, coverage, pregnancy, SARS-CoV-2, flu

## Abstract

**BACKGROUND:**

Pregnant women and their newborns are at increased risk of severe outcomes from influenza and COVID-19 infections; maternal vaccination is recommended. However, no routine surveillance of maternal vaccine coverage exists in Norway.

**AIM:**

To provide insights into vaccination coverage and timing during pregnancy.

**METHODS:**

This population-based registry study included women who gave birth in Norway during 1 October 2023–30 September 2024. Data on influenza and COVID-19 vaccinations administered during 1 October 2023–10 May 2024 were obtained from the national immunisation registry and linked to birth data from the Medical Birth Registry Norway. Cumulative coverage included vaccines administered during pregnancy, with sub-analyses focusing on second and third trimester vaccinations, month of delivery and maternal age.

**RESULTS:**

Overall influenza vaccination coverage was 29.9% (15,915/53,161), with 22.3% (11,856/53,161) vaccinated in the second or third trimester. Coverage increased from 16.4% (7,287/44,454) in October to 26.4% (12,982/49,170) in November and plateaued thereafter. Coverage peaked among women delivering in February (50.8%; 2,159/4,248) and declined afterwards. COVID-19 vaccination coverage was 12.1% (6,423/53,161) with 10.1% (5,349/53,161) in the second or third trimester, following a similar pattern to influenza. Overall, 11.4% received both vaccines. The lowest uptake (< 19%) was among women aged 25 years or younger.

**CONCLUSION:**

Coverage of maternal influenza and COVID-19 vaccinations for 2023/24 remained low, with missed opportunities to reach pregnant women beyond November 2023. Overall, the coverage was lowest among women aged 25 years or younger. Strengthened efforts are needed to increase vaccination coverage among pregnant women and reduce gaps in protection.

**Key public health message**

**What did you want to address in this study and why?**

Vaccination against influenza and COVID-19 is recommended during pregnancy, but there is limited information on how many pregnant women are actually vaccinated. Using nationwide registry data from Norway, we examined how many pregnant women received influenza and COVID-19 vaccines during the 2023/24 influenza season, at what stage of pregnancy they received the vaccine, and whether uptake differed by age group or region.

**What have we learnt from this study?**

Among over 50,000 women, we found that coverage for influenza and COVID-19 vaccines was low during the 2023/24 influenza season, at 29.9% and 12.1% respectively. Coverage varied substantially by month of delivery and timing during pregnancy, with missed vaccination opportunities for women beyond autumn, at the start of the influenza season and those aged 25 years or younger.

**What are the implications of your findings for public health?**

Our findings highlight the importance of timely monitoring of maternal vaccine coverage to inform targeted communication and interventions, particularly for groups with lower uptake, e.g. younger women. Clear recommendations are essential for protecting pregnant women and their newborns. Embedding vaccination into routine maternal health services could inform broader strategies to support improved and more equitable coverage across Europe.

## Introduction

Since the 1960s, the World Health Organization (WHO) has promoted maternal vaccination, leading to the widespread introduction of seasonal influenza, pertussis and more recently COVID-19 vaccines for pregnant women [1,2]. Influenza and SARS-CoV-2 infection during pregnancy have been associated with adverse maternal and neonatal outcomes. Influenza infection has been linked to an increased risk of stillbirth (relative risk: 3.62; 95% confidence interval (CI): 1.60–8.20) [3]. SARS-CoV-2 infection during pregnancy has also been linked to adverse pregnancy outcomes, including preterm birth and stillbirth, compared with uninfected pregnancies, with reported effect estimates indicating a moderate increase in risk but wide confidence intervals for stillbirth, highlighting the importance of vaccination in this group [4,5]. Although strong evidence supports the safety and efficacy for both mothers and their infants, coverage remains suboptimal and varies widely globally [6,7]. During the 2023/24 influenza season in the northern hemisphere, only eight of the 29 European Union/European Economic Area (EU/EEA) recommending maternal influenza vaccination reported coverage data, which ranged from 1% to 58% [8]. Between September 2023 and July 2024, just two countries, Spain (8%) and Ireland (17%), provided disaggregated COVID-19 vaccination coverage for pregnant women [9]. Inconsistent reporting, evolving definitions and limited access to data sources hinder international comparability and highlight the need for strengthened maternal vaccination surveillance.

In Norway, influenza vaccination has been recommended for pregnant women since 2009 and COVID-19 vaccination since August 2021. Both vaccines are generally advised during the second and third trimester, while administration in the first trimester is only advised for pregnant women with underlying health risk or occupational exposure [10]. The influenza vaccine is provided during the influenza season (Oct–May), whereas the COVID-19 vaccine is available year-round [11]. Currently, pregnant women in Norway pay between NOK 150 and 500 (approximately EUR 13–40, depending on municipality) for influenza vaccination, although the vaccine was provided free of charge during the 2021/22 season; COVID-19 vaccination continues to be free of charge. Women obtain vaccines mainly through self-initiated appointments with general practitioners, pharmacies or health clinics. Although these recommendations and access pathways have been in place for several years, Norway still lacks a national routine surveillance system for maternal vaccination coverage [12]. As both vaccines are generally advised for administration in the second and third trimesters, understanding the timing of vaccinations during pregnancy is essential for adjusting vaccination programmes.

This study aims to calculate the overall influenza and COVID-19 vaccination coverage among pregnant women, assess the timing of vaccinations during pregnancy, as well as regional and age-related differences. The findings can inform strategies to enhance maternal vaccination and strengthen surveillance across EU/EEA countries facing similar challenges.

## Methods

### Study setting, population and registries

This nationwide, population-based registry study was based on two Norwegian national registries (Supplementary Table S1, RECORD Checklist). Our study population comprised all women who delivered in Norway between 1 October 2023 and 30 September 2024, as identified in the Medical Birth Registry Norway (MBRN) [13]. Notification of all births and stillbirths from 22 weeks of gestation to the MBRN has been mandatory since 1967 and is routinely validated against the National Population Register [13].

Each resident in Norway has a unique national personal identification number (PIN), which enables linkage across national registries. Births to women without a PIN, such as tourists, unregistered individuals, or recently arrived migrants are not captured; this group accounts for fewer than 300 deliveries annually. Maternal vaccination data were obtained by linking MBRN records to the Norwegian Immunisation Registry (SYSVAK). Established in 1995 to monitor vaccines provided in the childhood vaccination programme [14]. Since January 2011, registration of all vaccinations for all age groups has been mandatory, with optional reporting of vaccinations administered abroad. Healthcare professionals who administer vaccines are responsible for accurate reporting. SYSVAK records the vaccination statuses, provides official national coverage statistics, and supports vaccine quality assurance and research through impact.

From the MBRN, we retrieved date of birth, gestational age at birth (in days), maternal age and county of residence. From SYSVAK, we retrieved codes for influenza and COVID-19 vaccination and the administration date for each vaccine. Details of included variables are provided in the Supplementary Table S2.

### Statistical analyses and definitions

We included all women registered in MBRN who delivered between 1 October 2023 and 30 September 2024. For the 2023/24 influenza season (1 Oct 2023–10 May 2024), the cumulative vaccination coverage for influenza and COVID-19 was calculated by including all women who received the vaccine at any point during their pregnancy (see more details Supplementary Text S1). The denominator for each month included all women who were pregnant during that month, based on the estimated date of conception and date of delivery reported in MBRN. We excluded cases with missing information on the days of gestation from MBRN and type of vaccination from SYSVAK from the analyses and used the woman/pregnancy as the unit of analysis. Sub-group analyses focused on second and third trimester vaccinations, because of the vaccine recommendations in Norway. In addition, we stratified vaccine uptake by month of delivery, gestational age and county of residence. Timing of vaccination during the observation period was categorised as: before pregnancy or during pregnancy in the first, second or third trimester. Vaccination in each category was summarised by month of delivery. We categorised maternal age at delivery in the following groups (< 20, 20–25, 26–30, 31–35, 36–40 and > 40 years). The place of residence was categorised by the county (NUTS level 3). The influenza season 2023/24 was grouped in early (Oct–Dec), mid (Jan–Feb) and late influenza season (Mar–May) [15]. To compute gestational age, we estimated the date of conception by subtracting the number of days of gestation at the time of delivery. The gestational age was also used to determine the trimester, defined based on the Norwegian Health Directorate, as first trimester (0–97 days), second trimester (98–195 days), and third trimester (195–300 days) [16]. All analyses were performed using R Version 4.3.0 and geographic variations in data were visually presented using the '*csmaps*' package in R [17].

## Results

We included a total of 53,161 women who were registered in the MBRN between 1 October 2023 and 30 September 2024 in Norway. The median age at delivery was 32 years (interquartile range (IQR): 29–35). The median number of births per month was 4,472 (IQR: 4,233–4,789), with the highest number of births recorded in the summer months of July 2024 (n = 4,861) and August 2024 (n = 4,921).

### Coverage and timing of influenza vaccination

**Figure 1:**
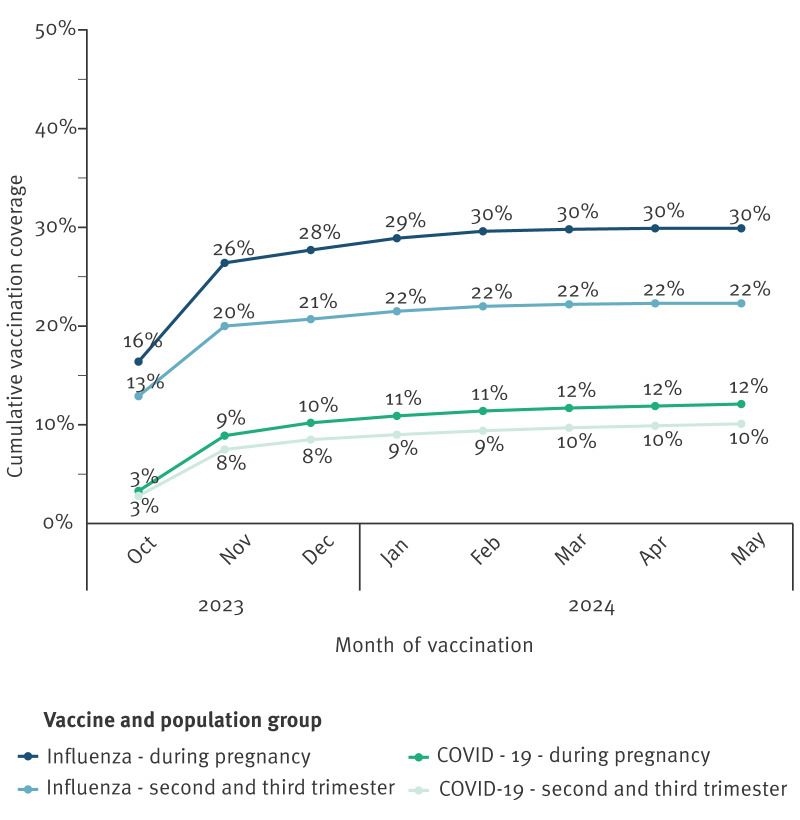
Influenza and COVID-19 vaccination coverage among women who delivered between 1 October 2023 and 30 September 2024 and were vaccinated between 1 October 2023–10 May 2024, Norway (n = 53,161)

The cumulative influenza vaccination coverage was 29.9% (15,915/53,161), and 22.3% (11,856/53,161) of the women were vaccinated in the second or third trimester. Most vaccinations were administered in October and November 2023, with overall coverage increasing from 16.4% (7,287/44,454) in October to 26.4% (12,982/49,170) in November, before plateauing (Figure 1). Vaccinations were most frequently administered during the second trimester (14–27 weeks gestation) in November and December, with widespread distribution across all gestational ages (an overview of influenza vaccines administered by gestational age is provided in Supplementary Figure S1). From December 2023 to May 2024, the total number of vaccinations per month decreased considerably.

Cumulative monthly coverage is displayed for women who received the vaccine at any point during pregnancy (influenza: dark blue and COVID-19: dark green) and those vaccinated in the second or third trimester (influenza: light blue and COVID-19: light green). Data on the women were extracted from the Medical Birth Registry Norway (MBRN) and data on the vaccines were extracted from the Norwegian Immunisation Registry (SYSVAK).

**Figure 2:**
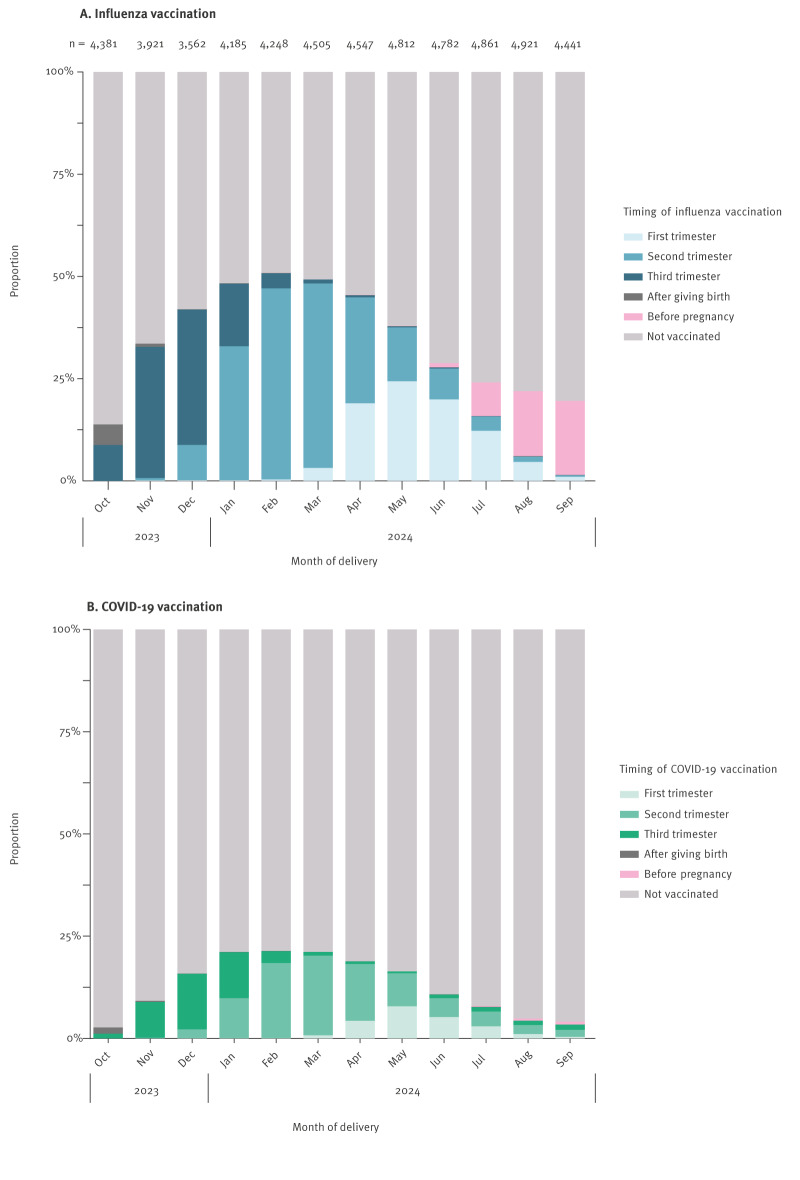
Proportion of influenza vaccination and COVID-19 vaccination by pregnancy stage and month of delivery, Norway, 1 October 2023–30 September 2024 (n = 53,161)

When estimating the coverage by month of delivery, we observed a gradual increase from 13.8% (604/4,381) in October to 50.8% (2,159/4,248) among those who delivered in February (Figure 2A). An overview of the proportion of women vaccinated against influenza by month of delivery and calendar month is provided in Supplementary Table S3). After February, the coverage decreased and was lowest among women who delivered in August and September. The timing of influenza vaccinations varied substantially by month of delivery. Those who delivered early in the influenza season (Oct–Nov 2023) were mostly vaccinated in their third trimester, whereas those who delivered later in the season (Apr–May 2024) were more often vaccinated in the second or first trimester (Figure 2A). The detailed influenza vaccination numbers during pregnancy (first, second, and third trimester), as well as before pregnancy and after giving birth, are provided in Supplementary Table S4. Pregnancies that ended outside the main vaccination period, i.e. in Jun–Sep 2024, showed a different vaccination pattern, with the majority of women receiving the vaccine before conception or in the early stages of gestation. Among those who delivered in August and September 2024, vaccination before pregnancy was observed, with a median time of 9 weeks (IQR: 4–14) between vaccination and conception.

Influenza (A) and COVID‑19 (B) vaccination by month of delivery (Oct 2023–Sep 2024), stratified by the timing of vaccination during pregnancy (first, second, and third trimester), as well as before pregnancy and after giving birth. The number of women eligible for vaccination each month (denominator) is shown above each bar (panel A). Denominators are identical for panels A and B.

### Coverage and timing of COVID-19 vaccination

The cumulative vaccination coverage for COVID-19 was 12.1% (6,423/53,161), and 10.1% (5,349/53,161) of the women were vaccinated in the second or third trimester. Most vaccinations were administered in October and November 2023. The overall coverage increased from 3.3% (1,454/44,454) in October to 10.2% (5,359/52,666) in December and plateaued thereafter (Figure 1). Most of the vaccines were administered during the second trimester (14–27 weeks gestation) in October and November 2023, with widespread distribution across gestational ages (an overview of COVID-19 vaccines administered by gestational age is provided in Supplementary Figure S2. From December 2023 to May 2024, the number of vaccinations per month decreased considerably.

When analysing coverage of COVID-19 vaccination by month of delivery, we observed a lower absolute number of vaccinations but similar distribution over time when compared with influenza vaccinations. The COVID-19 coverage increased gradually from women who delivered in October (2.7%; 116/4,381) and was highest among those delivering between January (21.1%; 884/4,185) and March (21.1%; 952/4,505). After March 2024, the coverage decreased among those who delivered later in the observation period (Figure 2B). An overview of the proportion of women vaccinated against COVID-19 by month of delivery and calendar month is provided in Supplementary Table S5.

The timing of COVID-19 vaccination during pregnancy by month of delivery followed a pattern similar to the influenza vaccination. Most women who delivered early in the season were vaccinated in their third trimester, whereas those delivering mid or late season, were vaccinated in their second trimester. Only women who delivered between July and October 2024 received the vaccine before pregnancy (Figure 2B). The detailed COVID-19 vaccination numbers during pregnancy (first, second, and third trimester), as well as before pregnancy and after giving birth, are provided in Supplementary Table S6, with a median of 8 weeks (IQR: 4–15) between vaccination and conception.

### Combined influenza and COVID-19 vaccine uptake 

Overall, 11.4% (6,053/53,161) of women received both the influenza and the COVID-19 vaccine during pregnancy. Among these, 8.0% (4,277/53,161) received both vaccines during the second or third trimester as recommended.

### Vaccination coverage differences among counties and age groups

**Figure 3:**
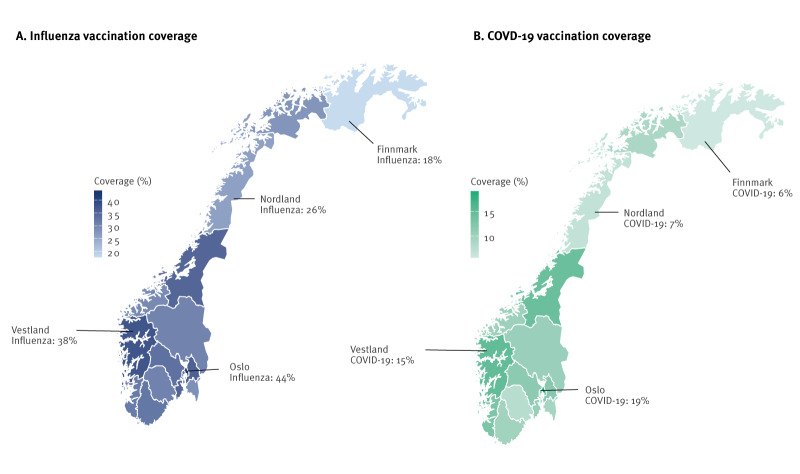
Influenza and COVD-19 vaccination coverage for counties in Norway, 1 October 2023–10 May 2024 (n = 53,161)

The highest coverage for both vaccines was identified among women living in the counties Oslo (Eastern Norway) and Vestland (Western Norway), and the lowest coverage was identified in Finnmark and Nordland (both in Northern Norway). Vaccination coverage varied by county for both vaccines, ranging from 18% to 44% for influenza and 6% to 19% for COVID-19 (Figure 3). The detailed vaccination coverage for influenza and COVID-19 for each county is provided in Supplementary Table S7). Among all age groups the vaccination coverage (< 20% for influenza and < 5% for COVID-19) was lowest among women aged 25 years or younger. The detailed vaccination coverage for influenza and COVID-19 for each age group is provided in Supplementary Table S8).

Influenza (A) and COVID-19 (B) vaccination coverage. The two counties with the highest and the two with the lowest coverage are labelled with percentages. Darker colours indicate higher coverage, and lighter colours indicate lower coverage.

## Discussion

Despite universal recommendations for vaccination during pregnancy, coverage in the influenza season 2023/24 was low, with 29.9% and 12.0% for influenza and COVID-19, respectively, while only 11.4% receiving both vaccines. Previous Norwegian data on pregnant women reported similar influenza coverage (27.7%) and higher COVID-19 coverage (31.8%) between September 2021 and December 2022 [12]. These findings highlight persistent gaps in maternal immunisation.

Most influenza vaccines were administered in October and November 2023 (Supplementary Figure S1 and Table S3), aligning with national trends where over 90% of influenza doses were administered by mid-November 2023 [18]. Coverage varied by month of delivery, reaching up to 50.8% for influenza vaccination in February 2024, while coverage was lower among women delivering later in the season. A similar decline in coverage later in the season was observed for COVID-19 vaccination (Supplementary Figure S2 and Table S5). The WHO defines a missed opportunity for vaccination as an eligible individual encountering healthcare services without being offered a vaccination despite no contraindications. In this context, women who attended antenatal care early in pregnancy but were not vaccinated at that timepoint represent potential missed opportunities, as they were rarely vaccinated later in pregnancy. Integrating vaccination offers into routine antenatal visits and initiating early discussions could help ensure every opportunity to protect both mother and child [19].

Ideally, influenza and COVID-19 vaccines should be administered towards the end of the second or beginning of the third trimester [20,21]. Vaccination later in pregnancy remains beneficial, as studies demonstrating effective maternal immune responses and placental antibody transfer [20,22]. For influenza, clinical protection is more closely linked to vaccination timing within the epidemic period than to gestational age, supporting continued promotion of vaccination throughout the season [23]. Strengthening consistent recommendations and maintaining awareness among antenatal healthcare providers could help improve coverage and timely protection for both vaccines.

The differences in vaccination coverage between influenza and COVID-19 in the 2023/24 season may reflect epidemiological and societal factors related to the unique dynamics of COVID-19. Until August 2021, COVID-19 vaccination during pregnancy in Norway was recommended only for women at high risk. After the general recommendation was introduced, coverage reached 54% among women delivering between May 2021 and May 2022 [24]. The lower coverage in 2023/24 likely reflects that most women had already completed COVID-19 vaccination in previous years and perceived this as sufficient protection during pregnancy [25]. In contrast, influenza coverage was also low in previous seasons (27.7% in 2021/22) [12], highlighting persistent challenges in seasonal influenza during pregnancy. For context, influenza uptake among the risk group of older adults (≥ 65 years) typically exceeds 67%, while coverage among adults aged 18–64 years is around 20–30% [18,26].

Survey data from Norway indicate generally positive attitudes towards vaccination, yet practical and psychological barriers, so-called ‘constraints’, remain key obstacles to uptake [27]. Structural factors such as co-payment requirements and limited administration during routine antenatal visits may further reduce coverage [28,29]. The potential of midwives who conduct most antenatal care to provide information on vaccination is likely underutilised, as payment systems and the limited integration of vaccination into routine services may limit opportunities for vaccine delivery [30,31].

International evidence supports integrating free vaccination into routine antenatal care. In France, a multicentre study demonstrated that providing influenza vaccination free of charge during antenatal consultations substantially increased vaccination coverage [32]. Removing financial barriers and embedding maternal vaccination in standard antenatal services could similarly improve coverage in Norway [32]. Following the introduction of the pertussis vaccine into Norway's maternal immunisation programme in May 2024, the vaccination coverage reached 73.0% within 12 months [33]. This high uptake likely reflects free vaccine provision and integration into routine antenatal care, an effect also observed in France [29,32]. It may additionally be influenced by individual risk perception, knowledge and confidence in different vaccines [34].

Despite the Norwegian population's generally high level of trust in health authorities and recommendations [35], our results show considerable regional differences in vaccination coverage, with lower rates in the northern health region and among women aged 25 years or younger. Similar geographic disparities have been reported for other vaccines, where coverage in northern Norway is often below the national average [36]. Research indicates that reliance on social media for health information among younger women and pregnant women is associated with higher vaccine hesitancy [37,38]. This highlights the need to explore information sources trusted by both unvaccinated and vaccinated women in more detail. In addition, accessibility and location of vaccination services in regions with lower coverage should be evaluated. These national patterns reflect broader international trends [39].

Internationally, influenza and COVID-19 vaccine uptake among pregnant women has varied widely and has generally remained below the WHO target of 75% for at-risk groups [39-41]. Comparisons across studies remain challenging because most countries lack comprehensive, population-based data, and estimates often rely on surveys or cohort studies with differing definitions and observation periods [42]. These factors highlight the need for harmonised definitions and standardised reporting of maternal vaccination coverage worldwide. Within Europe, similar challenges persist. Comprehensive surveillance systems for maternal vaccination are currently lacking across EU/EEA countries, and suboptimal uptake [8] combined with variable integration of vaccination into antenatal care further hinder systematic monitoring. Strengthening surveillance systems and embedding vaccination in routine maternal health services could inform broader strategies to improve coverage and equity across Europe.

Our study has several limitations. We could not analyse detailed sociodemographic characteristics, medical conditions or health factors. Only deliveries, not ongoing pregnancies, are registered in the MBRN, and quality checks require an additional 6–12 months, delaying data availability. This delay affects the timeliness of coverage surveillance, with figures for the 2023/24 season not available until spring 2025. This limits the value for real-time decision making and current-season public health efforts from such analyses. Another limitation when interpreting the results on the timing of vaccinations for different month of delivery is mainly influenced by the fixed influenza vaccination season rather than by individual decisions. In Norway, vaccines are typically available from October through May (the influenza season), with campaigns concentrated in October and November. This explains our observation why women delivering early in the season were usually vaccinated late in pregnancy, whereas those delivering later in the season were vaccinated earlier. These patterns are likely artefacts of seasonal vaccine availability and gestational timing rather than reflecting true differences in uptake behaviour [43]. Nevertheless, they highlight missed opportunities during and after the main campaign period and inform strategies to optimise timing and delivery of vaccination.

## Conclusion

Our findings reveal important gaps in maternal vaccination coverage in Norway, despite high public trust in health authorities. They emphasise the need for more targeted strategies and better integration of vaccinations into routine antenatal care. Similar challenges globally, including limited surveillance systems and inconsistent service integration, highlight the importance of coordinated efforts to strengthen maternal immunisation and promote its uptake across Europe and worldwide.

## Data Availability

The analysis code will be made available upon request. The data from this study cannot be shared due to data privacy regulations. However, data are available for research purposes upon request from the Norwegian Institute of Public Health, provided approval has been granted by the Norwegian Committee for Medical and Health Research Ethics in accordance with Norwegian data protection legislation.

## Supplementary Data

388000 Supplementary Material
