# Supplementary material for: Missed opportunities for maternal immunisation against influenza and COVID-19, Norway, October 2023 to May 2024: a population-based registry study
Source: Euro Surveill. 2026 Feb 19;31(7):2500504. doi: 10.2807/1560-7917.ES.2026.31.7.2500504 (PMC12924000; doi:10.2807/1560-7917.ES.2026.31.7.2500504)
Supplement: Supplementary Material [file 25-00504_STECHER_Supplement.pdf]

Supplementary Materials

This supplementary material is hosted by *Eurosurveillance* as supporting information alongside the article **Missed opportunities for maternal immunisation against influenza and COVID-19, Norway, October 2023 to May 2024: a population-based registry study**, on behalf of the authors, who remain responsible for the accuracy and appropriateness of the content. The same standards for ethics, copyright, attributions and permissions as for the article apply. Supplements are not edited by *Eurosurveillance* and the journal is not responsible for the maintenance of any links or email addresses provided therein.

Table S1. *The RECORD (Reporting of Studies Conducted using Observational Routinely-collected health Data) Statement checklist of items extended from the STROBE (Strengthening the Reporting of Observational Studies in Epidemiology) Statement, used to report observational studies using routinely collected health data.*

	**Item No.**	**STROBE items**	**Location in manuscript where items are reported**	**RECORD items**	**Location in manuscript where items are reported**
**Title and abstract**					
	1	(a) Indicate the study's design with a commonly used term in the title or the abstract (b) Provide in the abstract an informative and balanced summary of what was done and what was found		RECORD 1.1: The type of data used should be specified in the title or abstract. When possible, the name of the databases used should be included.<br><br>RECORD 1.2: If applicable, the geographic region and timeframe within which the study took place should be reported in the title or abstract.<br><br>RECORD 1.3: If linkage between databases was conducted for the study, this should be clearly stated in the title or abstract.	See title and abstract.<br><br>Abstract-methods and findings.<br><br>Abstract-methods and findings section.
**Introduction**					
Background rationale	2	Explain the scientific background and rationale for the investigation being reported	Introduction, second paragraph.		
Objectives	3	State specific objectives, including any prespecified hypotheses	Introduction, third paragraph.		
**Methods**					
Study Design	4	Present key elements of study design early in the paper	Methods, second paragraph.		
Setting	5	Describe the setting, locations, and relevant dates, including periods of recruitment, exposure, follow-up, and data collection	Methods, second paragraph.		

Participants	6	(a) Cohort study - Give the eligibility criteria, and the sources and methods of selection of participants. Describe methods of follow-up<br><br>Case-control study - Give the eligibility criteria, and the sources and methods of case ascertainment and control selection. Give the rationale for the choice of cases and controls<br><br>Cross-sectional study - Give the eligibility criteria, and the sources and methods of selection of participants<br><br>(b) Cohort study - For matched studies, give matching criteria and number of exposed and unexposed<br><br>Case-control study - For matched studies, give matching criteria and the number of controls per case		RECORD 6.1: The methods of study population selection (such as codes or algorithms used to identify subjects) should be listed in detail. If this is not possible, an explanation should be provided.<br><br>RECORD 6.2: Any validation studies of the codes or algorithms used to select the population should be referenced. If validation was conducted for this study and not published elsewhere, detailed methods and results should be provided.<br><br>RECORD 6.3: If the study involved linkage of databases, consider use of a flow diagram or other graphical display to demonstrate the data linkage process, including the number of individuals with linked data at each stage.	Methods, third paragraph, and S1 Table.<br><br>Discussion, limitations and strengths.<br><br>Methods and Text S2
Variables	7	Clearly define all outcomes, exposures, predictors, potential confounders, and effect modifiers. Give diagnostic criteria, if applicable.		RECORD 7.1: A complete list of codes and algorithms used to classify exposures, outcomes, confounders, and effect modifiers should be provided. If these cannot be reported, an explanation should be provided.	The list of included variables and outcome variables are listed in the Methods and Table S2.
Data sources/ measurement	8	For each variable of interest, give sources of data and details of methods of assessment (measurement).<br><br>Describe comparability of assessment methods if there is more than one group	Methods, paragraphs 2-3 and S2 Table.		
Bias	9	Describe any efforts to address potential sources of bias	Methods, paragraph 6-7.		
Study size	10	Explain how the study size was arrived at	Methods, paragraph 2		

Quantitative variables	11	Explain how quantitative variables were handled in the analyses. If applicable, describe which groupings were chosen, and why	Methods, paragraph 3		
Statistical methods	12	(a) Describe all statistical methods, including those used to control for confounding<br>(b) Describe any methods used to examine subgroups and interactions<br>(c) Explain how missing data were addressed<br>(d) Cohort study - If applicable, explain how loss to follow-up was addressed<br>Case-control study - If applicable, explain how matching of cases and controls was addressed<br>Cross-sectional study - If applicable, describe analytical methods taking account of sampling strategy<br>(e) Describe any sensitivity analyses	Methods, paragraph 2 & 3 and Text S1		
Data access and cleaning methods		..		RECORD 12.1: Authors should describe the extent to which the investigators had access to the database population used to create the study population.<br>RECORD 12.2: Authors should provide information on the data cleaning methods used in the study.	Methods, paragraph 2<br>Methods, paragraph 2-3, Text S1
Linkage		..		RECORD 12.3: State whether the study included person-level, institutional-level, or other data linkage across two or more databases. The methods of linkage and methods of linkage quality evaluation should be provided.	Methods paragraph 2, Text S1
**Results**					

Participants	13	(a) Report the numbers of individuals at each stage of the study (e.g., numbers potentially eligible, examined for eligibility, confirmed eligible, included in the study, completing follow-up, and analysed)<br><br>(b) Give reasons for non-participation at each stage.<br><br>(c) Consider use of a flow diagram		RECORD 13.1: Describe in detail the selection of the persons included in the study (i.e., study population selection) including filtering based on data quality, data availability and linkage. The selection of included persons can be described in the text and/or by means of the study flow diagram.	Methods, paragraph 1, Text S1
Descriptive data	14	(a) Give characteristics of study participants (e.g., demographic, clinical, social) and information on exposures and potential confounders<br><br>(b) Indicate the number of participants with missing data for each variable of interest<br><br>(c) Cohort study - summarise follow-up time (e.g., average and total amount)	Results, paragraph 1		
Outcome data	15	Cohort study - Report numbers of outcome events or summary measures over time<br><br>Case-control study - Report numbers in each exposure category, or summary measures of exposure<br><br>Cross-sectional study - Report numbers of outcome events or summary measures	Results, paragraph 2, and Figure xx		
Main results	16	(a) Give unadjusted estimates and, if applicable, confounder-adjusted estimates and their precision (e.g., 95% confidence interval). Make clear which confounders were adjusted for and why they were included<br><br>(b) Report category boundaries when continuous variables were categorized	n.a.		

		(c) If relevant, consider translating estimates of relative risk into absolute risk for a meaningful time period			
Other analyses	17	Report other analyses done—e.g., analyses of subgroups and interactions, and sensitivity analyses	Results, paragraph 3		
**Discussion**					
Key results	18	Summarise key results with reference to study objectives	Discussion, paragraph 1.		
Limitations	19	Discuss limitations of the study, taking into account sources of potential bias or imprecision. Discuss both direction and magnitude of any potential bias	Discussion, paragraphs 6-7, (Limitations)	RECORD 19.1: Discuss the implications of using data that were not created or collected to answer the specific research question(s). Include discussion of misclassification bias, unmeasured confounding, missing data, and changing eligibility over time, as they pertain to the study being reported.	Discussion, paragraphs 6-7.
Interpretation	20	Give a cautious overall interpretation of results considering objectives, limitations, multiplicity of analyses, results from similar studies, and other relevant evidence	Discussion, paragraphs 1-4.		
Generalisability	21	Discuss the generalisability (external validity) of the study results	Discussion, paragraph 6.		
**Other Information**					
Funding	22	Give the source of funding and the role of the funders for the present study and, if applicable, for the original study on which the present article is based	See funding statement		
Accessibility of protocol, raw data, and programming code		..		RECORD 22.1: Authors should provide information on how to access any supplemental information such as the study protocol, raw data, or programming code.	See data availability

Reference: Benchimol EI, Smeeth L, Guttmann A, Harron K, Moher D, Petersen I, Sørensen HT, von Elm E, Langan SM, the RECORD Working Committee. The REporting of studies Conducted using Observational Routinely-collected health Data (RECORD) Statement. PLoS Medicine 2015; in press.

Table S2. *Variables included from the Norwegian Immunisation Registry (SYSVAK) and the Medical Birth Registry Norway (MBRN) by variable and format of the variable.*

	Variables	Format
MBRN		
	Study ID mother / Personal identification number	9-digit code
	Date of birth mother	DD.MM.JJJJ
	County	name
	Date of birth child	DD.MM.JJJJ
	Gestational age	days calculated based on date of birth (days)
	Date of conception	calculated by date of birth and gestational age (DD.MM.JJJJ)
	Age at delivery (based on information from the National Population Register (Folkeregisteret)	Years
SYSVAK		
	Personal Identifier / Personal identification number	9-digit code
	Vaccine code	COVID-19 or Influenza
	Date of vaccination	DD.MM.JJJJ
	Vaccine Status	1= vaccinated, 0= not vaccinated

Text S1. Data quality and linkage of registry data.

Variables listed in Table S1 were obtained from the Medical Birth Registry of Norway (MBRN), established to monitor and improve maternal and child health, and to support research, public health surveillance, and quality assurance in maternity and neonatal care. All births from 22 weeks of gestation onwards are verified through linkage with the Norwegian Population Registry ("Folkeregisteret"). As the date of birth is embedded in the Norwegian personal identification number (PIN), birth data are complete and cross-validated with the population registry. County information is also routinely verified against the same source.

Vaccination data, including vaccine code, date of administration, and vaccination status, were retrieved from the Norwegian Immunisation Registry (SYSVAK), where reporting of all vaccinations is mandatory and subject to systematic quality control. The datasets were linked at the person-level using the unique PIN.

Text S2. Information on the statistical analysis of the cumulative influenza and COVID-19 vaccination coverage.

In this study, cumulative influenza and COVID-19 vaccination coverage was defined as the percentage of the target population (all women registered in the MBRN between 01 October 2023 and 30 September 2024) who received a vaccine between 01 October 2023 and 10 May 2024. The cumulative vaccination coverage was calculated using the following formula:

$$\text{Coverage}_{\text{cumulative}} = \frac{V}{P} \times 100\%$$

where, V = number of pregnant women vaccinated at any time during pregnancy within the study period (01 October 2023 - 10 May 2024) and P = total number of pregnant women registered in the MBRN between 01 October 2023 and 30 September 2024.

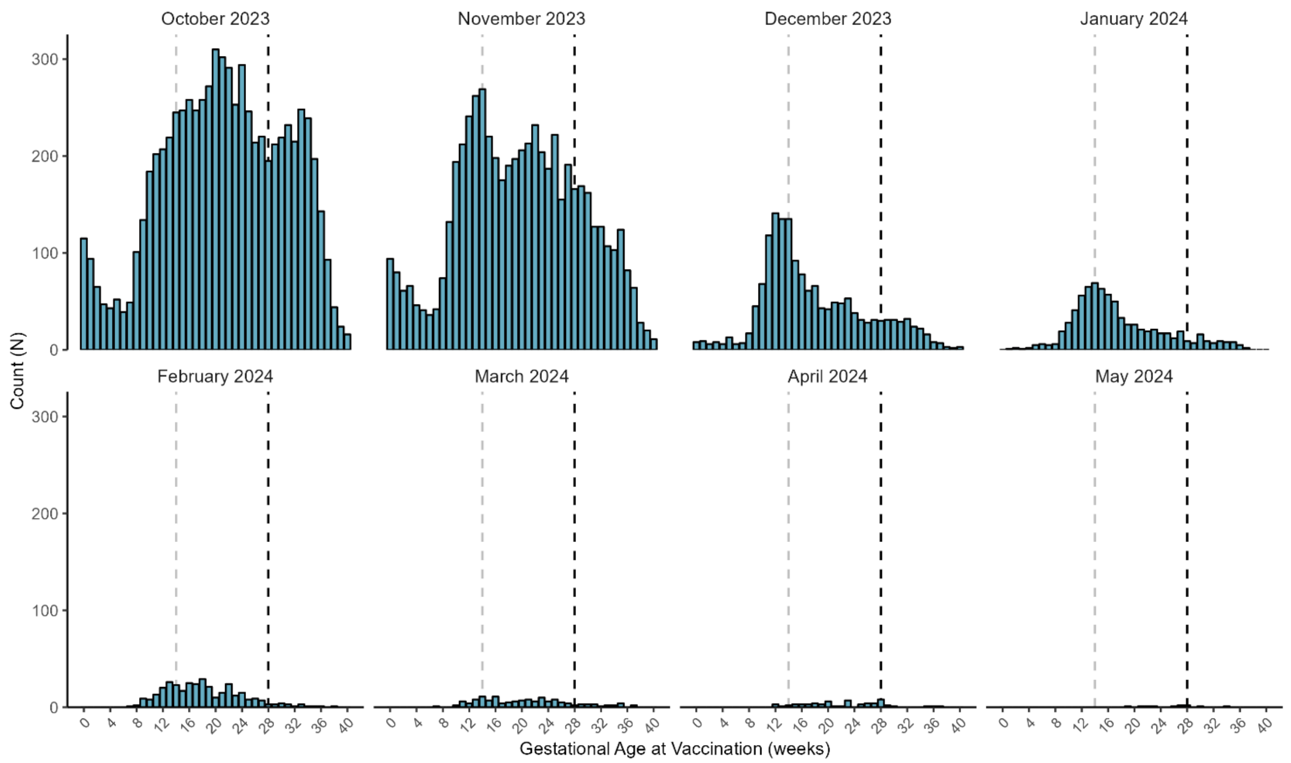

Figure S1: *Number of influenza vaccines administered by gestational age (week 0-40) between October 2023 and May 2024, among women registered in the Medical Birth Registry Norway (MBRN) who delivered between 1 October 2023 and 30 September 2024. The start of the second trimester (week 14) and third trimester (week 28) are indicated by grey and black dashed lines, respectively (N= 53,161).*

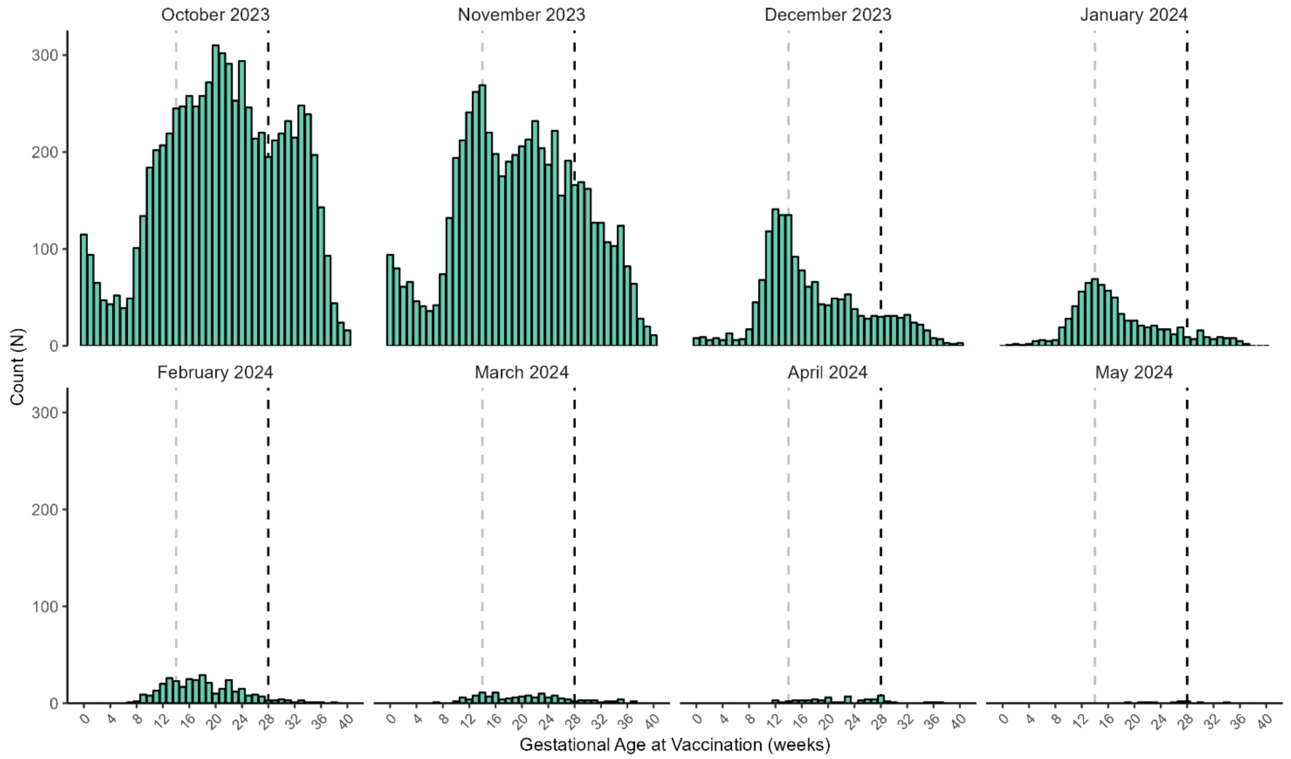

Figure S2: *Number of COVID-19 vaccines administered by gestational age (week 0-40) between October 2023 and May 2024, among women registered in the Medical Birth Registry Norway (MBRN) who delivered between 1 October 2023 and*

30 September 2024. The start of the second trimester (week 14) and third trimester (week 28) are indicated by grey and black dashed lines, respectively (N= 53,161).

Table S3. The proportion of women vaccinated against influenza by month of delivery and calendar month for the influenza season 2023–2024 in Norway (N = 53,161). Each woman was only included in the sample for the month in which she gave birth.

	Month of Delivery												
	**2023-10**	**2023-11**	**2023-12**	**2024-01**	**2024-02**	**2024-03**	**2024-04**	**2024-05**	**2024-06**	**2024-07**	**2024-08**	**2024-09**	
Month of Vaccination	n (%)	n (%)	n (%)	n (%)	n (%)	n (%)	n (%)	n (%)	n (%)	n (%)	n (%)	n (%)	Total Vaccinated by Month
2023-10	385 (8.8)	1056 (26.9)	943 (26.5)	1157 (27.6)	1168 (27.5)	1079 (24)	795 (17.5)	336 (7)	297 (6.2)	476 (9.8)	489 (9.9)	433 (9.8)	8614
2023-11	168 (3.8)	230 (5.9)	518 (14.5)	748 (17.9)	809 (19)	928 (20.6)	914 (20.1)	912 (19)	292 (6.1)	270 (5.6)	390 (7.9)	341 (7.7)	6520
2023-12	43 (1)	17 (0.4)	31 (0.9)	102 (2.4)	141 (3.3)	153 (3.4)	244 (5.4)	378 (7.9)	445 (9.3)	84 (1.7)	30 (0.6)	37 (0.8)	1705
2024-01	3 (0.1)	5 (0.1)	1 (0)	12 (0.3)	39 (0.9)	48 (1.1)	82 (1.8)	121 (2.5)	224 (4.7)	194 (4)	40 (0.8)	7 (0.2)	776
2024-02	1 (0)	2 (0.1)	-	1 (0)	2 (0)	6 (0.1)	20 (0.4)	59 (1.2)	75 (1.6)	95 (2)	69 (1.4)	12 (0.3)	342
2024-03	-	1 (0)	-	-	-	4 (0.1)	11 (0.2)	14 (0.3)	31 (0.6)	26 (0.5)	37 (0.8)	18 (0.4)	142
2024-04	-	-	-	-	-	-	1 (0)	2 (0)	11 (0.2)	19 (0.4)	16 (0.3)	13 (0.3)	62
2024-05	1 (0)	-	-	-	-	-	-	-	1 (0)	5 (0.1)	3 (0.1)	2 (0)	12
Total Vaccinated Among Deliveries	601	1311	1493	2020	2159	2218	2067	1822	1376	1169	1074	863	

Table S4. Influenza vaccination before, during pregnancy, and after pregnancy, by months of delivery, between October 2023 and September 2024.

	Month of Delivery											
	**2023-10**	**2023-11**	**2023-12**	**2024-01**	**2024-02**	**2024-03**	**2024-04**	**2024-05**	**2024-06**	**2024-07**	**2024-08**	**2024-09**
Total Deliveries	4381	3921	3561	4185	4248	4505	4549	4813	4782	4861	4920	4439
	n (%)	n (%)	n (%)	n (%)	n (%)	n (%)	n (%)	n (%)	n (%)	n (%)	n (%)	n (%)
Not vaccinated	3777 (86.2)	2605 (66.4)	2067 (58.0)	2163 (51.7)	2088 (49.2)	2287 (50.8)	2479 (54.5)	2989 (62.1)	3404 (71.2)	3691 (75.9)	3841 (78.1)	3572 (80.4)
Before pregnancy	-	-	-	-	1 (0)	-	3 (0.1)	3 (0.1)	50 (1)	399 (8.2)	782 (15.9)	805 (18.1)
After delivery	219 (5)	30 (0.8)	3 (0.1)	3 (0.1)	1 (0)	-	1 (0)	1 (0)	1 (0)	1 (0)	4 (0.1)	-
1st trimester	-	5 (0.1)	6 (0.2)	7 (0.2)	14 (0.3)	142 (3.2)	862 (19)	1173 (24.4)	953 (19.9)	597 (12.3)	227 (4.6)	45 (1)
2nd trimester	2 (0)	21 (0.5)	308 (8.6)	1372 (32.8)	1987 (46.8)	2034 (45.1)	1179 (25.9)	634 (13.2)	362 (7.6)	169 (3.5)	66 (1.3)	17 (0.4)
3rd trimester	383 (8.7)	1260 (32.1)	1178 (33.1)	640 (15.3)	157 (3.7)	42 (0.9)	23 (0.5)	12 (0.2)	12 (0.3)	4 (0.1)	1 (0)	2 (0)

Table S5. The proportion of women vaccinated against COVID-19 *by month of delivery and calendar month for the influenza season 2023–2024 in Norway. Each woman was only included in the sample for the month in which she gave birth.*

	**Month of Delivery**												
	**2023-10**	**2023-11**	**2023-12**	**2024-01**	**2024-02**	**2024-03**	**2024-04**	**2024-05**	**2024-06**	**2024-07**	**2024-08**	**2024-09**	
Month of Vaccination	n (%)	n (%)	n (%)	n (%)	n (%)	n (%)	n (%)	n (%)	n (%)	n (%)	n (%)	n (%)	Total Vaccinated by Month
2023-10	51 (1.2)	226 (5.8)	208 (5.8)	267 (6.4)	269 (6.3)	231 (5.1)	156 (3.4)	36 (0.7)	9 (0.2)	11 (0.2)	11 (0.2)	6 (0.1)	1481
2023-11	39 (0.9)	124 (3.2)	319 (9)	504 (12)	492 (11.6)	515 (11.4)	479 (10.5)	390 (8.1)	74 (1.5)	28 (0.6)	15 (0.3)	16 (0.4)	2995
2023-12	23 (0.5)	5 (0.1)	36 (1)	105 (2.5)	110 (2.6)	152 (3.4)	140 (3.1)	236 (4.9)	175 (3.7)	21 (0.4)	5 (0.1)	7 (0.2)	1015
2024-01	1 (0)	3 (0.1)	-	7 (0.2)	34 (0.8)	34 (0.8)	48 (1.1)	64 (1.3)	120 (2.5)	103 (2.1)	7 (0.1)	-	421
2024-02	-	1 (0)	-	-	2 (0)	14 (0.3)	20 (0.4)	35 (0.7)	62 (1.3)	103 (2.1)	44 (0.9)	5 (0.1)	286
2024-03	1 (0)	-	-	-	-	6 (0.1)	12 (0.3)	14 (0.3)	26 (0.5)	30 (0.6)	38 (0.8)	15 (0.3)	142
2024-04	1 (0)	-	-	1 (0)	-	-	4 (0.1)	10 (0.2)	28 (0.6)	31 (0.6)	40 (0.8)	22 (0.5)	137
2024-05	-	-	-	-	-	-	-	3 (0.1)	12 (0.3)	29 (0.6)	18 (0.4)	24 (0.5)	86
Total Vaccinated Among Deliveries	116	359	563	884	907	952	859	788	506	356	178	95	

Table S6. COVID-19 vaccination before, during pregnancy, and after pregnancy, by months of delivery, between October 2023 and September 2024.

	**Month of Delivery**											
	**2023-10**	**2023-11**	**2023-12**	**2024-01**	**2024-02**	**2024-03**	**2024-04**	**2024-05**	**2024-06**	**2024-07**	**2024-08**	**2024-09**
Total Deliveries	4381	3921	3561	4185	4248	4505	4549	4813	4782	4861	4920	4439
	n (%)	n (%)	n (%)	n (%)	n (%)	n (%)	n (%)	n (%)	n (%)	n (%)	n (%)	n (%)
Not vaccinated	4265 (97.4)	3561 (90.8)	2998 (84.2)	3301 (78.9)	3340 (78.6)	3553 (78.9)	3688 (81.1)	4024 (83.6)	4267 (89.2)	4476 (92.1)	4683 (95.2)	4261 (95.9)
Before pregnancy	-	-	-	-	-	-	1 (0)	-	-	11 (0.2)	25 (0.5)	29 (0.7)
After delivery	65 (1.5)	10 (0.3)	1 (0)	1 (0)	1 (0)	-	-	-	2 (0)	1 (0)	-	-
1st trimester	-	6 (0.2)	78 (2.2)	410 (9.8)	781 (18.4)	879 (19.5)	632 (13.9)	388 (8.1)	221 (4.6)	178 (3.7)	108 (2.2)	75 (1.7)
2nd trimester	-	-	1 (0)	1 (0)	2 (0)	33 (0.7)	195 (4.3)	378 (7.9)	250 (5.2)	142 (2.9)	53 (1.1)	18 (0.4)
3rd trimester	51 (1.2)	344 (8.8)	484 (13.6)	472 (11.3)	124 (2.9)	40 (0.9)	31 (0.7)	22 (0.5)	42 (0.9)	53 (1.1)	52 (1.1)	58 (1.3)

Table S7. Geographic distribution of influenza and COVID-19 vaccination coverage among pregnant women, by county, October 2023–May 2024 (N = 53,161).

		Influenza		COVID-19	
**County**	**Total**	**Vaccinated (n)**	**Coverage (%)**	**Vaccinated (n)**	**Coverage (%)**
Agder	2953	741	25.1	274	9.3
Akershus	7049	1934	27.5	800	11.3
Buskerud	2321	601	26.0	252	11.0
Finnmark	710	125	17.6	40	5.6
Innlandet	2930	764	26.1	293	10.0
Møre og Romsdal	2428	620	25.8	234	9.6
Nordland	2095	535	25.5	136	6.5
Oslo	8390	3645	43.5	1553	18.5
Rogaland	5287	1374	26.1	645	12.2
Telemark	1472	380	25.8	107	7.3
Troms	1465	374	25.6	124	8.5
Trøndelag	4622	1220	26.4	619	13.4
Vestfold	2137	577	27.0	219	10.2
Vestland	6236	2344	37.6	902	14.5
Østfold	2667	681	25.6	225	8.4

Table S8. Influenza and COVID-19 vaccination coverage among pregnant women, by age group, October 2023–May 2024 (N = 53,161).

		Influenza		COVID-19	
**County**	**Total**	**Vaccinated (n)**	**Coverage (%)**	**Vaccinated (n)**	**Coverage (%)**
<20	120	17	14.2	5	4.2
20-25	3992	652	16.3	163	4.1
26-30	15186	4039	26.6	1351	8.9
31-35	21308	7205	33.8	3069	14.4
36-40	10095	3332	33.0	1527	15.1
>40	2075	623	30.0	287	4.2
Unknown	385	47	12.2	21	5.5
